# Association between PER and CRY gene polymorphisms and the response to caffeine citrate treatment in infants with apnea of prematurity

**DOI:** 10.3389/fped.2024.1414185

**Published:** 2024-07-22

**Authors:** Jiang-Biao Xie, Wei Zhuang, Yao Zhu, Zhi Zheng, Yan-Ru Huang, Si-Min Ma, Xin-Zhu Lin

**Affiliations:** ^1^ Department of Neonatology, Women and Children’s Hospital, School of Medicine, Xiamen University, Xiamen, China; ^2^ Xiamen Key Laboratory of Perinatal-Neonatal Infection, Xiamen, China; ^3^ Department of Pharmacy, Women and Children’s Hospital, School of Medicine, Xiamen University, Xiamen, China; ^4^ Department of Central Laboratory, Women and Children’s Hospital, School of Medicine, Xiamen University, Xiamen, China

**Keywords:** apnea of prematurity, caffeine, gene polymorphism, circadian rhythm, *PER* gene, *CRY* gene

## Abstract

**Background:**

Circadian rhythms impact metabolism and the therapeutic effects of drugs. The purpose of this study was to determine the association between *PER* and *CRY* polymorphisms and caffeine citrate treatment response in infants with apnea of prematurity.

**Methods:**

A total of 221 preterm infants of gestational age <34 weeks were included in this study (160 in the response group and 61 in the non-response group). The propensity score matching method was used to perform a 1:1 matching for all premature infants, and the general characteristics and clinical outcomes of the two groups were compared. The association between polymorphisms of the circadian transcription repressors *PER* and *CRY* and caffeine citrate treatment response in infants with apnea of prematurity was analyzed with co-dominant, dominant, recessive, and over-dominant models, as well as analysis of alleles. Generalized multifactor dimensionality reduction (GMDR) analysis was used to analyze the interaction between the *PER* and *CRY* genes.

**Results:**

After propensity score matching, 45 preterm infants were included in each of the response and non-response groups, and there were no statistically significant differences in general characteristics between the two groups (*P *> 0.05). Infants in the non-response groups had a higher incidence of moderate and severe bronchopulmonary dysplasia (BPD) (*P *= 0.043), retinopathy of prematurity (ROP) (*P *= 0.035), and invasive ventilation (*P *= 0.027), and their duration of oxygen use (*P *= 0.041) was longer. When corrected for false discovery rate, the *PER3* rs228669 recessive model (*P*_FDR_*_ _*= 0.045) and the over-dominant model (*P*_FDR_ = 0.045) were both associated with caffeine citrate treatment response. Preterm infants with the rs228669 CC genotype had a significantly lower rate of caffeine citrate non-response in the recessive model (OR = 0.28, 95% CI = 0.12–0.66), which was significantly higher in preterm infants with the CT genotype in the over-dominant model (OR = 4.18, 95% CI = 1.64–10.66). GMDR analysis revealed an interaction between the *PER* and *CRY* genes (*P *< 0.05).

**Conclusions:**

Circadian rhythms may play a role in the response of premature infants to caffeine citrate, and polymorphisms of the *PER* and *CRY* genes may influence the effectiveness of caffeine citrate treatment for apnea of prematurity.

## Introduction

Apnea of prematurity (AOP), resulting from an immature brainstem and abnormal respiratory control in preterm infants, is a common phenomenon in the neonatal intensive care unit (NICU). AOP incidence has a negative correlation with gestational age, ranging in prevalence from 20% to 85% in infants born between 30 and 34 weeks of gestation, and reaching 100% for those born before 28 weeks (1). Caffeine citrate is the drug most commonly used to treat AOP, given its long half-life, high bioavailability, and minimal side effects, providing both short- and long-term clinical benefits (2). However, some preterm infants respond poorly to it despite adequate dosing (3–5). Its efficacy has been reported to be closely related to genetic factors as well as the infant's degree of development and the dose administered (6).

Recently, there has been increasing interest in environmental and temporal rhythmic cues in the NICU, including light cycles, temperature variations, sound levels, feeding rhythms, and timing of medication supply, and it has been recognized that these factors may impact newborn outcomes (7). Circadian rhythms, arising from natural selection during long-term evolution, aid in maintaining a variety of physiological functions in plants and animals and in regulating their adaptation to the internal and external environment. Disruption of circadian rhythms alters physiologic functions of human tissues and organs and disease occurrence, and affects drug pharmacokinetics within the body (8, 9).

The primary molecular mechanism generating circadian rhythms arises from the transcription-translation-feedback loop formed by the cyclic expression of circadian genes. There are four core clock components: the transcription activators circadian locomotor output cycles protein kaput (CLOCK) and brain and muscle arnt-like 1(BMAL1), and the transcription repressors cryptochrome (CRY) and period circadian protein (PER) (10). Caffeine delays the human circadian clock *in vivo* and *in vitro*, altering circadian rhythm phases (11) and affecting the sleep-wake cycle in preterm infants (12). *CLOCK* gene polymorphisms have been found to influence preterm infant response to caffeine citrate. This finding suggests that circadian rhythm genes may function in determining the clinical efficacy of caffeine citrate therapy for AOP (13). The transcription repressors PER and CRY are equally important for the formation and alternation of circadian rhythms, but their role in caffeine citrate response for AOP has not been determined. Therefore, this study sought to determine the impact of *PER* and *CRY* gene polymorphisms on the efficacy of caffeine citrate for treatment of AOP.

## Methods

### Study population

This prospective case-control study included premature infants cared for between October 2021 and June 2023 in the NICU of Women and Children's Hospital, School of Medicine, Xiamen University. Our study adhered to the principles of the Helsinki Declaration and was approved by the Ethics Committee of the Women and Children's Hospital, School of Medicine, Xiamen University (Approval No.: KY-2020-040).

Inclusion criteria: preterm infants born during this period at a gestational age of <34 weeks who were administered a standard dose of caffeine citrate within 24 h of birth for the treatment or prevention of AOP.

Exclusion criteria: Infants with (1) early onset sepsis (EOS); (2) grade III-IV intraventricular hemorrhage (IVH); (3) severe congenital malformations or chromosomal abnormalities; (4) failure to obtain umbilical cord blood samples or insufficient DNA concentration in umbilical cord blood; (5) discharge against medical advice based on parental decision.

### Data collection

For the included premature infants, general characteristics, including gestational age, birth weight, sex, mode of delivery, completion of antenatal corticosteroid therapy, small for gestational age (SGA), prolonged rupture of membranes (>18 h), 5-min Apgar score, patent ductus arteriosus (PDA), and neonatal respiratory distress syndrome (NRDS), and clinical treatment outcomes were collected. Clinical treatment outcomes included: moderate and severe bronchopulmonary dysplasia (BPD), retinopathy of prematurity (ROP), stage II or greater necrotizing enterocolitis (NEC), duration of oxygen use, use of invasive ventilation, reintubation after extubating, and duration of caffeine citrate administration.

### Diagnostic criteria

(1) AOP: preterm infants (<37 weeks of gestational age) with cessation of breathing ≥20 s, or less than 20 s with decreased heart rate (<100 beats/min) and/or oxygen saturation (<85%) (1); (2) completion of antenatal corticosteroid therapy: two intramuscular doses of 12 mg of betamethasone to the mother over a 24-h period; (3) SGA: birth weight lower than the tenth percentile of infants of the same sex and gestational age, according to the 2013 Fenton curve; (4) PDA: persistence of a PDA for more than 72 h after birth, confirmed by echocardiography; (5) moderate (II) and severe (III) BPD: diagnosis and staging based on the National Institute of Child Health and Human Development (NICHD) diagnostic and grading criteria (2018) (14).; (6) stage II or greater NEC: diagnosis and staging based on the Bell criteria (15); (7) ROP: diagnosis according to the International Classification of Retinopathy of Prematurity, third edition (16); (8) NRDS: diagnosis according to the European Consensus Guidelines on the Management of Respiratory Distress Syndrome, 2022 (17); (9) EOS: blood or cerebrospinal fluid culture obtained within 72 h after birth with growth of a pathogenic bacterial species (18); (10) IVH: diagnosed and graded according to Papile et al. (19).

### Treatment and group

All preterm infants included in this study received appropriate respiratory support according to their condition on admission and were administered a standard dose of caffeine citrate within 24 h of admission: an intravenous loading dose of 20 mg/kg (1 ml:20 mg, containing 10 mg of caffeine and 10 mg of citrate, approved by the National Medical Products Administration with registration number H20163401 and manufactured by Chengdu Yuandong Biopharmaceutical Co., Ltd.) followed by a maintenance dose of 5 mg/kg/d. Caffeine citrate was switched to oral dosing once preterm infants achieved full enteral feeding (oral intake of 150 ml/kg/day). Caffeine citrate was discontinued once a corrected gestational age of 33–35 weeks was achieved and no AOP had occurred for 5–7 days. Based on the presence of AOP after caffeine citrate treatment, they were divided into non-response and response groups.

Definition of non-response: AOP occurring ≥2 times/day or once requiring bag and mask ventilation with supplemental oxygen after administration of the standard regimen within 3 d. Non-response preterm infants were given an additional dose of caffeine citrate at 5–10 mg/kg and the daily maintenance dose was increased to 10 mg/kg. If AOP persisted after this treatment, oxygen therapy was increased based on the infant's condition. Preterm infants who did not experience the above conditions were classified into the response group.

### Genotype analysis

At birth of preterm infants, one ml of umbilical artery blood was collected in an EDTA anticoagulation tube and stored at −80 °C. DNA was extracted using the QIAamp DNA Blood Mini Kit (Qiagen, USA) according to the manufacturer's instructions, and DNA concentration was measured using a UV spectrophotometer (Thermo Fisher Scientific, USA). SNPs located in the 5' flanking sequence, 5' untranslated region (UTR), exons, introns, and 3' UTR of the *PER* and *CRY* genes with a minor allele frequency (MAF) ≥ 0.05 for East Asian populations were selected using the dbSNP database (https://www.ncbi.nlm.nih.gov/). The final selection included six SNPs for genotyping: *CRY1* rs1056560, *CRY2* rs1401419, *PER1* rs2585405, *PER2* rs934945, *PER3* rs228669, and *PER3* rs2640908. Genotyping was performed using the Agena Sequenom MassArray Analyzer 4 system (Matrix-Assisted Laser Desorption/ionization Time-Of Flight). Primer information is provided in the Supplementary Table S1.

### Statistical analysis

Statistical analysis was conducted using SPSS V25.0. The Kolmogorov-Smirnov test was used to test normal distribution of continuous variables. Normally distributed samples are described using`*X *± S and compared using the independent samples t-test, and non-normally distributed samples are described using the median (quartiles) and compared using the Mann-Whitney U rank sum test. Categorical variables are expressed as numbers and percentages and were compared using the *χ*^2^ test. A logistic regression model was established using gestational age as the independent variable and response to treatment with caffeine citrate as the dependent variable. Subsequently, 1:1 nearest neighbor matching was performed using a caliper value of 0.2 times the log standard deviation of propensity scores. SNP analyses were performed using co-dominant, dominant, recessive, and over-dominant models as well as alleles. A logistic regression analysis was used to calculate the odds ratio (OR) and 95% confidence interval (CI), and false discovery rate (FDR) correction was performed to reduce type I errors using the Benjamini-Hochberg procedure. Using GMDR Software Beta V 0.7, GMDR analysis was employed to explore the association between SNP interaction and the efficacy of caffeine citrate treatment for AOP. *P *< 0.05 was considered statistically significant.

## Results

### General characteristics and clinical outcomes

**Figure 1:**
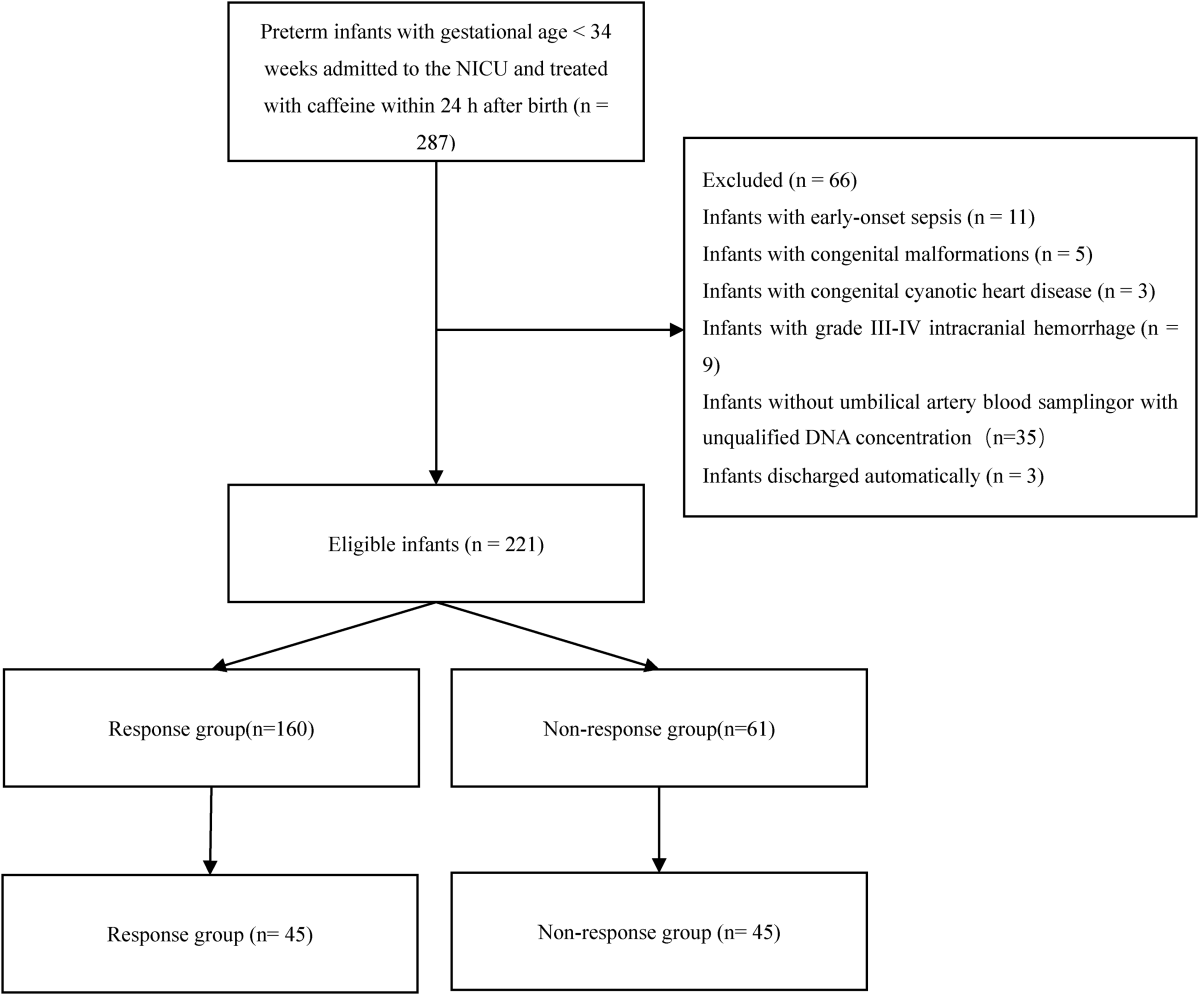
The flowchart of the study.

A total of 287 preterm infants were treated for or given prophylactic therapy to prevent AOP within 24 h of birth using a standard dose of caffeine citrate during the study period, and 221 preterm infants were finally included after applying the exclusion criteria. The study enrolment process is shown in Figure 1.

Among the 221 preterm infants included, there were 160 (72.4%) infants in the response group and 61 (27.6%) infants in the non-response group. Statistical differences were identified between the two groups in gestational age, weight, 5 min Apgar score (*P *< 0.05). For clinical treatment outcomes, there were significant differences in PDA, and RDS, moderate and severe BPD, ROP, duration of oxygen use, invasive ventilation, reintubation after extubating, and duration of caffeine citrate use (*P *< 0.005).

Propensity score matching resulted in 45 preterm infants in both the response and non-response groups. There were no statistically significant differences (*P *> 0.05) in general characteristics between the two groups. Of clinical treatment outcomes, the non-response group had a higher incidence of moderate and severe BPD (*P *= 0.035), ROP (*P *= 0.035), and invasive ventilation (*P *= 0.027), as well as a longer duration of oxygen use (*P *= 0.041). The differences in the incidence of reintubation after extubating, NEC grade II or greater, and duration of caffeine citrate administration were not statistically significant (*P *> 0.05). Details are presented in Table 1.

**Table 1:** General characteristics and clinical outcomes.

Variables	Non-response group (*n* = 61)	Response group (*n* = 160)	t/z/*χ*	*P*	Non-response group (*n* = 45)	Response group (*n* = 45)	t/z/χ	*P*
General characteristics								
Gestational age (w)	30.0 (28.2, 32.3)	31.04 (32.6, 33.4)	−5.446	**<0**.**001**	31.1 ± 1.9	31.1 ± 1.9	−0.061	0.952
Birth weight (g)	1,313.0 (1,016.5, 1,652.5)	1,742.5 (1,479.3, 1,965.0)	−5.454	**<0**.**001**	1,475.6 ± 381.4	1,592.6 ± 433.2	−1.359	0.178
Male *n* (%)	32 (52.5)	91 (56.9)	0.349	0.555	23	32	3.787	0.052
Cesarean section *n* (%)	45 (73.8)	106 (66.3)	1.154	0.283	35	29	1.947	0.163
Completed antenatal steroids *n* (%)	38 (62.3)	91 (56.9)	0.534	0.465	28	28	0.000	1.000
Premature rupture of membranes >18 h *n* (%)	19 (31.1)	36 (22.5)	1.767	0.184	14	16	0.200	0.655
SGA	7 (11.5)	12 (7.5)	0.888	0.346	6	2	0.195	0.138
5 min Apgar score M (Q1, Q3)	9 (8,9)	9 (9,10)	−3.388	**0**.**001**	9 (9, 10)	9 (9, 9)	−0.355	0.722
Clinical outcomes								
PDA *n* (%)	31 (50.8)	38 (23.8)	15.07	**<0**.**001**	19	12	2.411	0.120
NRDS *n* (%)	26 (42.6)	38 (23.8)	7.646	**0**.**006**	14	14	0.000	1.000
Moderate and severe BPD *n* (%)	21 (34.4)	13 (8.1)	23.468	**<0**.**001**	13	5	4.444	**0**.**035**
ROP *n* (%)	40 (65.6)	51 (31.9)	20.705	**<0**.**001**	27	17	4.447	**0**.**035**
Stage II or greater NEC	1 (1.6)	4 (2.5)	0.000	1.000^a^	1	1	0.000	1.000^a^
duration of oxygen use (d)	39.5 (22.6, 52.8)	16.0 (8.9, 28.3)	−6.273	**<0**.**001**	35.6 (14.7, 44.2)	20.4 (9.6, 37.8)	−2.047	**0**.**041**
Invasive ventilation *n* (%)	18 (29.5)	22 (13.8)	18.346	**<0**.**001**	12	4	4.865	**0**.**027**
Reintubation after extubating *n* (%)	4 (6.6)	1 (0.6)	4.602	**0**.**032**^a^	2	1	0.000	1.000^a^
duration of caffeine citrate use (d)	30.5 (17.0, 43.0)	12.5 (8, 21)	−6.147	**<0**.**001**	24.0 (12.5, 33.8)	18 (10, 31.5)	−0.789	0.430

SGA, small for gestational age; PDA, patent ductus arteriosus; NRDS, neonatal respiratory distress syndrome; BPD, bronchopulmonary dysplasia; NEC, necrotizing enterocolitis; ROP, retinopathy of prematurity.

The table on the left shows the data for the initial group, while the table on the right shows the data for the matched group.

^a^ Continuous corrected chi-square test; t: Statistical value of the independent samples t-test; z: Statistical value of the Mann-Whitney U rank sum test; χ:Statistical value of the chi-square test.

Bold values indicate statistically significant differences between group comparisons (*P* < 0.05).

### Association of PER and CRY gene polymorphisms with response to caffeine citrate in preterm infants

Table 2 summarizes the comparison of genotypes and genetic models of the PER and CRY genes between the response and non-response groups. The genotype frequencies of the *PER* and *CRY* gene SNPs all conformed to the Hardy-Weinberg equilibrium (*P *> 0.05). The *PER3* rs228669 allele frequency differed significantly between the groups (*P *< 0.05). Preterm infants with the rs228669 C allele had an increased rate of non-response to caffeine citrate (OR = 2.16, 95% CI = 1.11–4.20, *P *= 0.021). However, this result was not statistically significant after FDR correction (*P*_FDR_*_ _*> 0.05).

**Table 2:** Association of *PER* and *CRY* gene polymorphisms with response to caffeine citrate in preterm infants.

SNPs	Model	Genotype	Non-response group (*n* = 45)	Response group (*n* = 45)	OR (95% CI)	*P*	*P*_FDR_
*CRY1*rs1056560	Co-dominant	CC	4	3	1	0.082	0.205
		AC	10	20	0.38 (0.07–2.01)		
		AA	31	22	1.06 (0.22–5.20)		
	Dominant	CC	4	3	1	0.694	0.771
		AC/AA	41	42	0.70 (0.15–3.48)		
	Recessive	CC/AC	14	23	1	0.054	0.205
		AA	31	22	2.32 (0.98–5.47)		
	Over-dominant	CC/AA	35	25	1	0.025	0.125
		AC	10	20	0.36 (0.14–0.89)		
	Allele	C	18	26	1	0.165	0.291
		A	72	64	1.63 (0.82–3.24)		
*CRY2* rs1401419	Co-dominant	TT	25	33	1	0.184	0.306
		CT	18	10	2.38 (0.94–6.03)		
		CC	2	2	1.32 (0.17–10.02)		
	Dominant	TT	25	33	1	0.078	0.205
		CT/CC	20	12	2.20 (0.91–5.33)		
	Recessive	TT/CT	43	43	1	1.000	1.000
		CC	2	2	1.00 (0.14–7.43)		
	Over-dominant	TT/CC	27	35	1	0.069	0.205
		CT	18	10	2.33 (0.93–5.87)		
	Allele	T	68	76	1	0.136	0.287
		C	22	14	1.76 (0.83–3.70)		
*PER1* rs2585405	Co-dominant	CC	9	10	1	0.273	0.390
		GC	27	20	1.50 (0.51–4.38)		
		GG	9	15	0.67 (0.20–2.26)		
	Dominant	CC	9	10	1	0.796	0.853
		GC/GG	36	35	1.42 (0.42–3.15)		
	Recessive	CC/GC	36	30	1	0.153	0.287
		GG	9	15	0.50 (0.19–1.30)		
	Over-dominant	CC/GG	18	25	1	0.14	0.287
		GC	27	20	1.88 (0.81–4.33)		
	Allele	C	45	40	1	0.455	0.569
		G	45	50	0.80 (0.45–1.44)		
*PER2* rs934945	Co-dominant	CC	18	24	1	0.307	0.400
		TC	22	19	1.54 (0.65–3.67)		
		TT	5	2	3.33 (0.58–19.18)		
	Dominant	CC	18	24	1	0.205	0.324
		TC/TT	27	21	1.71 (0.74–3.96)		
	Recessive	CC/TC	40	43	1	0.238	0.357
		TT	5	2	2.69 (0.49–14.64)		
	Over-dominant	CC/TT	23	26	1	0.525	0.630
		TC	22	19	1.31 (0.57–3.01)		
	Allele	C	58	67	1	0.145	0.287
		T	32	23	1.61 (0.85–3.05)		
*PER3* rs228669	Co-dominant	TT	5	5	1	0.006	0.060
		CT	23	9	2.56 (0.59–11.00)		
		CC	17	31	0.55 (0.14–2.17)		
	Dominant	TT	5	5	1	1.000	1.000
		CT/CC	40	40	1.00 (0.27–3.72)		
	Recessive	TT/CT	28	14	1	0.003	**0**.**045**
		CC	17	31	0.28 (0.12–0.66)		
	Over-dominant	TT/CC	22	36	1	0.002	**0**.**045**
		CT	23	9	4.18 (1.64–10.66)		
	Allele	T	33	19	1	0.021	0.125
		C	57	71	0.46 (0.24–0.90)		
*PER3* rs2640908	Co-dominant	CC	7	11	1	0.061	0.205
		TC	31	20	2.44 (0.81–7.33)		
		TT	7	14	0.79 (0.21–2.92)		
	Dominant	CC	7	11	1	0.292	0.398
		TC/TT	38	34	1.76 (0.61–5.04)		
	Recessive	CC/TC	38	31	1	0.081	0.205
		TT	7	14	0.41 (0.15–1.14)		
	Over-dominant	CC/TT	14	25	1	0.019	0.125
		TC	31	20	2.77 (1.17–6.56)		
	Allele	C	45	42	1	0.655	0.756
		T	45	48	0.88(0.49–1.57)		

Bold values indicate statistically significant differences between group comparisons (*P* < 0.05).

In genetic model analysis, the genotype frequency differences between the response and non-response groups were statistically significant in the over-dominant model of *CRY1* rs1056560, the over-dominant model of *PER3* rs2640908, as well as the co-dominant, over-dominant, and recessive models of *PER3* rs228669 (*P *< 0.05). *CRY1* rs1056560 in the over-dominant model (OR = 0.36, 95% CI = 0.14–0.89, *P *= 0.025) and *PER3* rs228669 in the recessive model (OR = 0.28, 95% CI = 0.12–0.66, *P *= 0.003) were associated with a decreased rate of non-response to caffeine citrate treatment in preterm infants. Conversely, *PER3* rs228669 in the over-dominant model (OR = 4.18, 95% CI = 1.64–10.66, *P* = 0.002) and PER3 rs2640908 in the over-dominant model (OR = 2.77, 95% CI = 1.17–6.56, *P* = 0.019) were associated with an increased rate of non-response to caffeine citrate treatment. After FDR correction, the *PER3* rs228669 recessive model (*P*_FDR_* *= 0.045) and the over-dominant model (*P*_FDR_* *= 0.045) remained associated with caffeine citrate treatment response. The rate of non-response to caffeine citrate was significantly lower in preterm infants with the rs228669 CC genotype in the recessive model (OR* *= 0.28, 95% CI = 0.12–0.66) and significantly higher in preterm infants with the CT genotype in the over-dominant model (OR = 4.18, 95% CI = 1.64–10.66). None of the remaining SNPs showed any association in any genetic models (*P *> 0.05).

### Association of gene-gene interactions of PER and CRY in response to caffeine citrate in preterm infants

**Figure 2:**
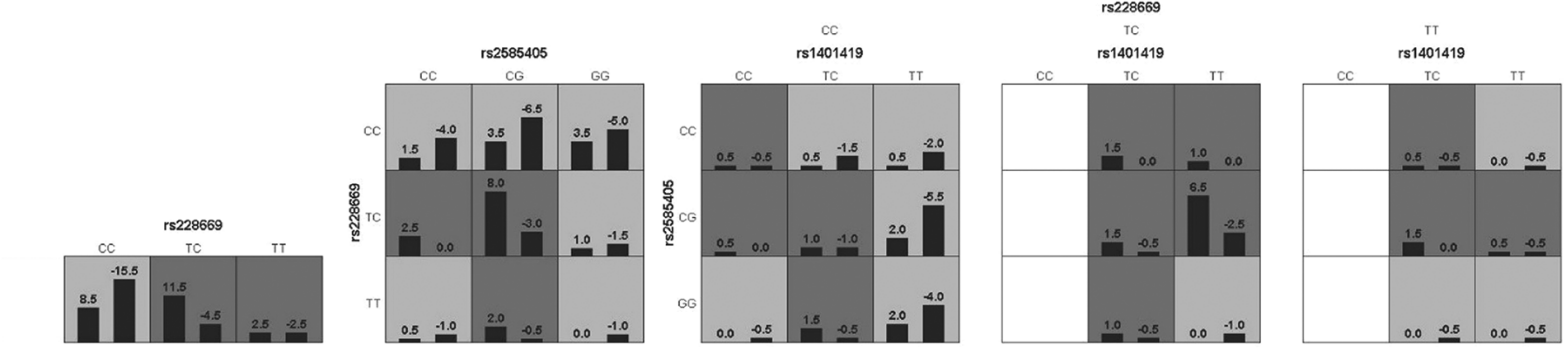
Gene interactions of *PER* and *CRY.*

GMDR analysis identified an interaction between the *PER* and *CRY* genes (Table 2 and Figure 2). The optimal SNP–SNP interaction models contained two and three genetic variants. The two-SNP model included *PER* rs228669 and *PER* rs2585405, with a cross-validation consistency of 9/10, a test accuracy of 0.6225, and a sign test of *P *= 0.0107. The three-SNP model included *PER* rs228669, *PER* rs2585405 and *CRY2* rs1401419, with a cross-validation consistency of 7/10, a test accuracy of 0.6300, and a sign test of *P *= 0.0010 (Table 3).

**Table 3:** Association of gene-gene interactions of *PER* and *CRY* with response to caffeine citrate in preterm infants.

The best model	Training accuracy	Testing accuracy	Sign test (P)	Cross-validation consistency
rs228669	0.6593	0.6225	9 (0.0107)	10/10
rs228669-rs2585405	0.7013	0.6225	9 (0.0107)	9/10
rs1401419-rs228669-rs2585405	0.7519	0.6300	10 (0.0010)	7/10

## Discussion

This study investigated the relationship between polymorphisms of the circadian transcription repressors *PER* and *CRY*, as well as their interactions, and the efficacy of caffeine citrate in treating AOP. After correction for multiple tests, the *PER3* rs228669 polymorphism was found to be significantly associated with caffeine citrate response in preterm infants in both the recessive and over-dominant models (*P*_FDR_* *< 0.05). Specifically, preterm infants with the rs228669 CC genotype had a significantly lower rate of non-response to caffeine citrate in the recessive model (OR = 0.28, 95% CI = 0.12–0.66), and preterm infants with the CT genotype had a significantly higher rate of non-response to caffeine citrate in the over-dominant model (OR = 4.18, 95% CI = 1.64–10.66). In addition, GMDR analyses indicated that gene-gene interactions of the *PER* and *CRY* genes may increase the risk of preterm infants being non-responsive to caffeine citrate treatment (*P *< 0.05).

Caffeine citrate has been used to treat AOP in preterm infants for half a century, but considerable individual variation exists in preterm infant response, with some responding poorly despite adequate caffeine citrate dosing. Long et al. (6) reported that poor response may be associated with genetic factors, particularly polymorphisms in the adenosine receptor, aromatic hydrocarbon receptor, adenosine deaminase, and phosphodiesterase genes. Preterm infants with poor responses to treatment tend to have a worse prognosis, including longer duration of oxygen use and hospitalization, and an increased risk of invasive ventilation and death (3–5). On one hand, the increased duration of oxygen use and the use of invasive ventilation may lead to a higher risk of BPD and ROP. On the other hand, although caffeine may reduce the incidence of BPD through its anti-inflammatory effect, however, this effect may also be related to genetic factors. Kumral et al. (3) reported that the incidence of BPD was also significantly increased in preterm infants with adenosine receptor genotype that results in non-response to caffeine citrate. Our results showed an increase in the duration of oxygen administration (*P *< 0.05) and in the incidence of invasive ventilation, BPD, and ROP in the non-response group of preterm infants (*P *< 0.05), which is consistent with published reports. It seemed vital, then, to explore the genetics of the response to caffeine citrate treatment for AOP. Caffeine, as a potent adenosine receptor antagonist, not only delays central circadian rhythms, but also enhances circadian clock sensitivity to light (20). Both acute and long-term caffeine use can alter sleep homeostasis (21, 22). Recently, Guo et al. (13) reported that *CLOCK* gene polymorphisms involved in the expression of the CLOCK-BMAL1 heterodimers impact treatment of AOP with caffeine citrate, confirming for the first time that circadian genes are associated with the response of AOP to caffeine citrate treatment. Circadian rhythms are regulated by the central clock of the hypothalamic suprachiasmatic nucleus (SCN), which synchronizes with the external environment by receiving light signals from the retina and transmitting temporal information to nearly all of the body's cells (23). At the molecular level, at the beginning of a circadian rhythm cycle, CLOCK and BMAL1 form heterodimers in the cell nucleus and bind to the enhancer box elements (E-boxes) of the upstream promoter of the *PER* and *CRY* genes, initiating their transcription and translation. PER and CRY similarly form heterodimers and accumulate in the cytoplasm, subsequently translocating to the nucleus and repressing their own transcription by inhibiting BMAL1-CLOCK heterodimer activity, creating a negative feedback loop with a period of approximately 24 h (11). Circadian rhythms can influence the metabolic processes of drugs and affect the efficacy and toxicity of drugs based on the timing of administration (24). *CLOCK* gene polymorphisms are closely associated with circadian preference, sleep duration, and sleep disorders, and affect the efficacy of caffeine citrate in AOP treatment (13, 25). As two core transcription repressors, PER and CRY also play a vital role in regulating circadian rhythms. Therefore, we speculated that *PER* and *CRY* gene polymorphisms might impact caffeine citrate efficacy in treating AOP.

As we speculated, we found that the *PER3* rs228669 polymorphism was significantly associated with AOP caffeine citrate treatment response in both the recessive and over-dominant models (*P*_FDR_* *< 0.05), with a significantly higher rate of caffeine citrate non-response in preterm infants with the CT genotype (OR = 4.18, 95% CI = 1.64–10.66), while preterm infants with the CC genotype had a significantly lower rate of non-response (OR = 0.28, 95% CI = 0.12–0.66). As an important gene regulating circadian rhythms, variants in the *PER3* gene are predominantly associated with the occurrence of sleep disorders (26). SNPs rs228669 in *PER3* gene are located in the exonic splicing enhancer (ESE) region, and the variant is synonymous. The SNP is located in the splice donor site and may alter the mRNA sequence, leading to either variations in protein structure or altered translation efficiency, thereby impacting the biological function of PER3. The rs228669 SNP affects cell metabolism and proliferation processes (27, 28). Our study identified a significant correlation between rs228669 and the response of premature infants to caffeine citrate, suggesting that the *PER3* polymorphism may impact the efficacy of caffeine citrate treatment for AOP by altering circadian rhythms.

Due to the interconnection of proteins encoded by the various clock genes, the combination of polymorphisms in these genes may influence phenotype. GMDR analysis showed a statistically significant interaction between *PER3* rs228669 and *PER1* rs2585405 (*P* = 0.0107). *PER1* rs2585405 is a missense variant, which changes the type and sequence of amino acids in the polypeptide chain. The rs2585405 missense variant is associated with susceptibility to noise-induced hearing loss and to prostate cancer in Chinese populations (29, 30). However, there have been no studies reporting how rs2585405 affects circadian rhythms. Future investigations may focus on the interaction between rs228669 and rs2585405 and how these impact circadian rhythms. In addition, the interactions among *PER3* rs228669, *PER1* rs2585405, and *CRY2* rs1401419 also showed statistical significance (*P *= 0.0010). Premature infants carrying the rs228669 TC, rs2585405 CG, and rs1401419 TT had the highest rate of non-response to caffeine citrate. This observation suggested that while CRY didn't directly impact the caffeine citrate response in preterm infants, it might have interacted with the PER gene, and this interaction collectively determined the efficacy of caffeine citrate for AOP. The *CRY1* and *CRY2* genes are highly expressed in the SCN, and their mRNA levels show a cyclic oscillation pattern. The circadian period is shortened when the *CRY1* gene is lacking, whereas it is lengthened when the *CRY2* gene is lacking (31). Combined polymorphisms of the *PER* and *CRY* genes may alter the inhibitory effect of the PER-CRY heterodimers on the activity of CLOCK-BMAL1 heterodimers, thereby altering circadian rhythms and potentially influencing caffeine citrate treatment efficacy.

Our study has some limitations. Firstly, although we ensured the homogeneity of general characteristics of premature infants between the two groups through propensity score matching, other factors such as external stimuli, potential complications, and the NICU environment might also influence the response to caffeine citrate in preterm infants, we didn't take them into account. Secondly, we only studied a limited number of SNPs of the *PER* and *CRY* genes, the findings may not fully capture the genetic complexity of circadian genes involved in premature infants' response to caffeine citrate. Thirdly, the subjects of this study were limited to a single-center population in China, so the applicability of the study's conclusions to other populations may be limited. Finally, our study's findings need further external validation in independent cohort. In conclusion, our study found that *PER* and *CRY* gene polymorphisms were genetic factors influencing premature infants' response to caffeine citrate, suggesting that circadian rhythms may impact the efficacy of caffeine citrate therapy for AOP. While few investigations have been conducted on this subject, future studies may explore temporal variations for caffeine citrate based on this evidence, such as adjusting the timing and frequency of medication dosing according to circadian rhythms. Additionally, interventions to promote the maturation and stability of premature infants' circadian rhythms through appropriate external stimuli such as light, temperature, sound, and touch to enhance the efficacy of caffeine citrate treatment also merit investigation (32). Our future research will be conducted both at the *in vivo* and *in vitro* levels and aim to uncover the underlying mechanisms to further enhance the precision of caffeine citrate therapy for AOP.

## Data Availability

The data presented in the study are deposited in the Figshare repository. The data is available here: https://doi.org/10.6084/m9.figshare.26304802
