# Supplementary material for: Association between PER and CRY gene polymorphisms and the response to caffeine citrate treatment in infants with apnea of prematurity
Source: Front Pediatr. 2024 Jul 22;12:1414185. doi: 10.3389/fped.2024.1414185 (PMC11301747; doi:10.3389/fped.2024.1414185)
Supplement: Supplementary file 1 [file Table1.pdf]

Gene	SNP	Forward primer (5' to 3')	Reveres primer (5' to 3')	Extension primer
CRY1	rs1056560	ACGTTGGATGCAAATTCTCTTGCCAAGTTC	ACGTTGGATGTCTAATGACATTTCTGTGG	TAATGAATTTCACAGAG
CRY2	rs1401419	ACGTTGGATGACCTCCAGACTAAGGATCG	ACGTTGGATGAACACTCCTGAGGCCAATAG	gggaTGAGAGAGTCAGGAA
PER1	rs2585405	ACGTTGGATGGAGCTGCATCTCGAGTTGAA	ACGTTGGATGACTCCCCTTCTCCATCCTTG	ctggaGGTGAGGAGGACTCGGGG
PER2	rs934945	ACGTTGGATGTTACGTCTGCTCTTCGATCC	ACGTTGGATGTTCTCTGGGACTCAGCGAAG	tgaagAAGAAGACGAAAATG
PER3	rs228669	ACGTTGGATGAGGAGCAGCTTGTCAGCATC	ACGTTGGATGATGTGACATAAGCCCATGC	ggggaCTCCAGTGAGGCCAG
PER3	rs2640908	ACGTTGGATGAATGGCAGTGAGAGCAGTC	ACGTTGGATGGTAGGATGGGATGGATTCTC	AGCAGTCCTGCTACTAC

Supplementary Table 1 The primer information of genes
